# Non-Cross Resistant Sequential Single Agent Chemotherapy in First-Line Advanced Non-Small Cell Lung Cancer Patients: Results of a Phase II Study

**DOI:** 10.1155/2009/457418

**Published:** 2009-11-12

**Authors:** V. Surmont, J. G. J. V. Aerts, K. Y. Tan, F. Schramel, R. Vernhout, H. C. Hoogsteden, R. J. van Klaveren

**Affiliations:** ^1^Department of Pulmonology, Erasmus MC-Daniel Den Hoed Cancer Center, Rotterdam, The Netherlands; ^2^Department of Pulmonology, Amphia Hospital, Breda, The Netherlands; ^3^Department of Pulmonology, St. Franciscus Gasthuis, Rotterdam, The Netherlands; ^4^Department of Pulmonology, St. Antonius, Nieuwegein, The Netherlands; ^5^Department of Trials and Statistics, Erasmus MC-Daniel Den Hoed Cancer Center, Rotterdam, The Netherlands

## Abstract

*Background*. sequential chemotherapy can maintain dose intensity and preclude cumulative toxicity by increasing drug diversity. *Purpose*. to investigate the toxicity and efficacy of the sequential regimen of gemcitabine followed by paclitaxel in first line advanced stage non-small cell lung cancer (NSCLC) patients with good performance status (PS). *Patients and methods*. gemcitabine 1250 mg/m^2^ was administered on day 1 and 8 of course 1 and 2; Paclitaxel 150 mg/m^2^ on day 1 and 8 of course 3 and 4. Primary endpoint was response rate (RR), secondary endpoints toxicity and time to progression (TTP). *Results*. Of the 21 patients (median age 56, range 38–80 years; 62% males, 38% females) 10% (2/21) had stage IIIB, 90% (19/21) stage IV, 15% PS 0, 85% PS 1. 20% of patients had a partial response, 30% stable disease, 50% progressive disease. Median TTP was 12 weeks (range 6–52 weeks), median overall survival (OS) 8 months (range 1–27 months), 1-year survival was 33%. One patient had grade 3 hematological toxicity, 2 patients a grade 3 peripheral neuropathy. *Conclusions*. sequential administration of gemcitabine followed by paclitaxel in first line treatment of advanced NSCLC had a favourable toxicity profile, a median TTP and OS comparable with other sequential trials and might, therefore, be a treatment option for NSCLC patients with high ERCC1 expression.

## 1. Introduction

Even with the use of novel chemotherapeutic agents, the prognosis of patients with advanced NSCLC remains poor. Platinum-based chemotherapy combined with either gemcitabine, vinorelbine, paclitaxel, or docetaxel is currently the mainstay in the treatment of advanced NSCLC [1–5]. Standard therapy for advanced NSCLC results in response rates of 20% to 40%, a median survival between 8–10 months, and 1-year survival rates between 30% and 50% [1–3].

Chemotherapy may lead to the selection of chemo-resistant tumor clones. Frequent exposure to different cytotoxic agents with brief intervals may inhibit tumor regrowth and limit the emergence of drug resistant cell lines [6, 7]. Sequential chemotherapy administration offers the possibility to increase drug diversity while maintaining dose intensity, potentially leading to less dose reductions, an optimal dose intensity, and prolonged treatment duration and disease control [6, 7].

In order to investigate the validity of this approach, we decided to conduct a nonrandomized phase II study, to investigate the toxicity and efficacy in terms of time to progression and response rate of a sequential single agent regimen consisting of gemcitabine followed by paclitaxel in the first-line treatment of patients with stage IIIB/IV NSCLC.

## 2. Patients and Methods

In this multicenter trial patients with stage IIIB (malignant pleural effusion or N3 due to supraclavicular lymph node involvement) and stage IV have been enrolled between 2003 and 2006. The study was approved by the ethical committees of the Erasmus MC and 4 other hospitals. Patients were included after written informed consent. Other selection criteria were measurable disease according to the RECIST criteria [8], age over 18 years, WHO performance status less than 2, adequate bone marrow reserve (absolute neutrophil count ≥ 2.0 × 10^9^/L, platelet count ≥ 100 × 10^9^/L), adequate hepatic function (total bilirubin ≤ 1.5 × upper normal limit, ASAT and ALAT ≤ 3.0 × upper normal limit, alkaline phosphatase ≤ 2.5 × upper normal limit, total billirubin 1.5–2.5 × upper normal limit and ASAT or ALAT 3–5 × upper normal limit in case of liver metastases).

Exclusion criteria were prior treatment with chemotherapy and the presence of other malignancies (previous or present), except adequately treated in situ carcinoma of the cervix or basal cell carcinoma of the skin and a previous malignancy more than 5 years ago without evidence of recurrence (except for malignant melanoma, hypernephroma, or breast cancer).

## 3. Treatment

**Figure 1:**
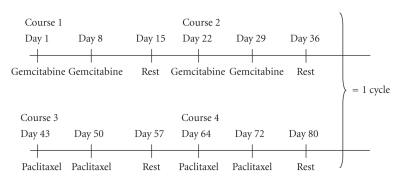
Treatment schedule.

Gemcitabine 1250 mg/m^2^ was administered intravenously on days 1 and 8 of courses 1 and 2 as a 30-minute infusion. Paclitaxel 150 mg/m^2 ^ was administered intravenously on days 1 and 8 of courses 3 and 4 as a 3-hour infusion. One course was defined as two weekly doses of chemotherapy followed by one week of rest and one cycle as 2 courses of gemcitabine followed by 2 courses of paclitaxel (Figure 1). At least 1 cycle was administered unless patient refusal or excessive toxicity precluded further therapy. If there was no PD after 1 cycle the same treatment schedule could be repeated up to a maximum of 2 cycles. If PD was observed at the end of the first or second cycle, further treatment was according to local policy.

## 4. Efficacy and Tolerability Assessments

Study assessments included physical examination, complete blood count, electrocardiogram, tumor measurements (chest *X*-ray, and chest-upper abdomen computed tomography scan), within 4 weeks before start of the treatment. Routine blood test for blood chemistry and haematological toxicity were performed before each chemotherapy administration. Response evaluation by CT took place after every 2 courses by RECIST criteria [8]. Toxicity was graded according to the National Cancer Institute Common Toxicity Criteria version 3 (NCI-CTC) and was assessed every 3 weeks by physical examination, direct questioning, and haematological and biochemical parameters.

## 5. Statistical Considerations

It was hypothesized that if the sequential regimen had an efficacy lower than 25%, it was unlikely to be of interest and would not result in further investigation. According to Fleming's single stage procedure P0 was set at 0.20. The response percentage that would certainly warrant further investigation (P1) was set at 0.40. With a power of 0.93 and an *α* of 0.06 this implies that 35 patients had to be enrolled. For the power calculation the best response during the first cycle has been used. *P*-values < .05 were considered significant.

## 6. Results

Twenty-one patients have been enrolled in this trial over a 3-year period due to competing trials in the participating centres. Median age was 56 years (range 38–80 years), 62% (13/21) was male, 38% (8/21) female, 10% (2/21) had stage IIIB, 90% (19/21) stage IV, 15% (2/21) ECOG performance status 0, 85% (18/21) ECOG 1. Ten (47.5%) patients had an adenocarcinoma, 3 (14.5%) squamous cell carcinoma, and 8 (38%) had large cell carcinoma.

One non-evaluable patient died one week after the first gemcitabine dose administration due to a cerebral vascular accident. At that time the platelet count was normal. Of the 20 evaluable patients 20% (4/20) achieved a partial response (PR), 30% (6/20) stable disease (SD), and 50% (10/20) progressive disease (PD). Five patients (25%) progressed after 2 courses of gemcitabine, all of them had an adenocarcinoma. Median time to progression (TTP) was 12 weeks (range 6–52 weeks), the median overall survival (OS) 8 months (range 1–27 months), and the 1-year survival rate 33%. Toxicity was mild; only one patient developed grade 3 hematological toxicity, in two others there was grade 3 peripheral neuropathy occurring at the second cycle. There were no treatment-related deaths. No dose reductions were needed.

## 7. Discussion

In this non-randomised phase II study, the sequential administration of single agent gemcitabine followed by paclitaxel in the first line treatment of advanced NSCLC had a favourable toxicity profile, a median TTP, and OS comparable with other sequential trials reported in the literature (Table 1) [9–20].

When we designed our study (2002-2003) Vansteenkiste et al. reported that treatment of patients with symptomatic advanced NSCLC with single agent gemcitabine resulted in a superior clinical-benefit response rate compared to cisplatin-based combination chemotherapy. Gemcitabine was equally effective in controlling “disease-specific” symptoms, but superior in controlling “constitutional” symptoms [21]. Therefore, single agent gemcitabine in first line treatment of stage IIIB/IV NSCLC was at that time a valid therapeutic choice and we decided to investigate the sequential administration of two single agent non-cross resistant chemotherapeutic drugs, gemcitabine, and paclitaxel. In the present study, paclitaxel was selected as sequential agent because taxanes do not require the presence of an intact p53 pathway for apoptosis induction in contrast to DNA-damaging agents like gemcitabine [22]. The dose of paclitaxel of 150 mg/m^2 ^ was based on the results of phases I and II trials [23–25]. Akerley et al. reported on a phase I trial of weekly paclitaxel administered over 3 hours for 6 consecutive weeks followed by 2 weeks of rest. From this study the recommended phase II dose was 175 mg/m^2^/week [23]. In the subsequent phase II trial this dose-dense regimen led to a high proportion of grade 3-4 neutropenia and grade 2-3 peripheral neuropathy (32%) [24]. Therefore, in the CALBG study 9713, weekly paclitaxel at a reduced dose of 150 mg/m^2^/week for 6 consecutive weeks was used followed by 2 weeks of rest. They demonstrated that this dose-dense regimen could be administered safely [25].

Because 50% of the participants in our trial had disease progression and because disease progression occurred already after 2 courses of single agent gemcitabine, we decide to close our study prematurely. At that time, it had also become evident from the literature that single agent gemcitabine in first line treatment of stage IIIB/IV NSCLC was inferior compared to platinum-based doublets [26, 27].

Even though our study has been closed prematurely, our data add to our current understanding on the treatment of NSCLC because they contribute to the concept of maintenance therapy with non-cross resistant drugs. In a recent randomized phase III trial of maintenance pemetrexed plus best supportive care versus placebo plus best supportive care, PFS was 4 months in the pemetrexed arm versus 2 months in the placebo arm with an hazard ratio (HR) of 0.6 (*P* < .00001) and the OS was 13.4 months versus 10.6 months, respectively (HR 0.79, *P* = .012) [28]. The phase III trial of immediate versus delayed docetaxel after first line chemotherapy in advanced NSCLC showed also a superior progression free survival (statistically significant) and greater median overall survival (not statistically significant) for the arm with immediate docetaxel [29]. These trials support the rationale of using a non-cross-resistant third generation agents before disease progression has occurred. We also believe that a single agent nonplatinum approach could be of value in ERCC1 positive patients, especially in the perspective of individualized treatment. Patients with completely resected NSCLC and ERCC1-negative tumors appeared to benefit from adjuvant cisplatin-based chemotherapy, whereas patients with ERCC1-positive tumors did not [30]. A prospective trial of 366 patients, in which patients with low ERCC1 were selected for platinum-based therapy (docetaxel, cisplatin), while those with high ERCC1 expression were directed to alternate non-platinum therapy (docetaxel, gemcitabine), demonstrated a significantly higher overall response rate in the genotypic arm compared to the non-selected control arm [31]. The response rate in the low ERCC1 group receiving platinum chemotherapy was 53%, but the RR in the high ERCC1 non-platinum arm was 47%, compared to 39% for the non-selected group receiving platinum therapy. A prospective phase II feasibility trial in which patient's therapy was selected based on ERCC1 expression showed that the low ERCC1 group treated with gemcitabine/carboplatin and the high ERCC1 group treated with gemcitabine/docetaxel had a similar median survival of 13 months and response rates of 44% [32].

Our data also confirm that gemcitabine is less active in adenoarcinoma's than that in squamous cell carcinoma's [1] because all patients in our trial who progressed after 2 courses of gemcitabine had an adencocarcinoma.

In conclusion, although this non-randomised phase II study failed to meet the primary efficacy endpoint, the sequential administration of single agent gemcitabine followed by paclitaxel in first line treatment of advanced NSCLC had a favourable toxicity profile, a median TTP, and OS comparable with other sequential trials reported in the literature and might, therefore, be a treatment option for NSCLC patients with high ERCC1 expression.

## Figures and Tables

**Table 1:** Overview of the sequential studies reported in the literature.

Study	Phase	*N* =	Regimen	RR (%)	PD(%)	MS (m)	1year OS (%)	PFS (m)	Major grade 3-4 toxicity(%)
Doublet → Doublet									

Gebbia (9)	III	400	G + IFO (2) → CDDP + VNR (2) versus	19	59	NR	NR	3.1^a^	Neutropenia 57/67/26/21 Thrombo 32/35/17^b^/41 Vomiting 13/15/21/23 Asthenia 29/35/35^c^/50
			CDDP + VNR (2) → G + IFO (2) versus	32	33	NR	NR	5	
			CDDP + VNR (up to 6 cycles) versus	44^a^	25	9	24	4.1	
			CDDP + G (up to 6 cycles)	34	37	8.2	20	4	

Doublet → SA									

Edelman (10)	III	204	CBDCA + G (3) → PTX(3) versus	21	29	9	34	4	Neutropenia 47^a^/70 Anemia 19/14
			CDDP + VNR (3) → DOC(3)	28	22	9	36	4	Thrombocytopenia 37/2^a^ Fatigue 7^a^/18 Emesis 5^a^/24 Toxic deaths 3/3
Clark (11)	II	18	CDDP (2) + VNR(2) → DOC (4)	31	44	9.5	44	NR	Leukopenia 45^d^ Emesis 26^d^ Toxic deaths 3
Grossi (12)	II	51	CDDP + PTX (2) → VNR( 2) → G (2)	43	25	14	53	6.8	Neutropenia 41 Toxic deaths 1
Kubota (13)	III	401	CBDCA + PTX (up to 6) versus	36^a^	10	13.8	55.5	6	Neutropenia 54/30^a^ Neuropathia 21/2^a^ Toxic deaths 0/2
			VNR + G (3) → DOC (3)	23	16	13.1	55.6	5.9	

SA → doublet or triplet									

Feliu (14)	II	52	PTX (6) → CDDP + GEM + VNR (up to 6)	56	31	NR	56	9	Neutropenia 20 Neuropathy 12 Emesis 10
Rixe (15)	II	32	DOC (4) → CDDP + VDS (4)	17	27	11	47	4.4	Neutropenia (gr 4) 71 Febrile neutropenia 14 Neuropathy 24

SA → SA									

Present study	II	21	G → PTX	20%	**50**	8	33	3	Neutropenia 4 Neuropathy 9
Manegold(16)	II-III	338	G +DOC (6) versus	33^a^	NR	7.3	27	6.3^a^	Neutropenia 36/27 Infection 17/13
			G (3) → DOC (3)	22	NR	7.4	25	4.9	Dyspnoe 21/20 Asthenia 12/11
Martoni (17)	II	52	G(3) → VNR (until PD)	23	23	10	42	6	Neutropenia 22^e^ Constipation 3^e^
Poon (18)	II	23	G (3) → CDDP (4)	21	52	14.6	63	3.3	Neutropenia 13 Anemia 13
Hirsch (19)	II	42	VNR (2) → G	38	36	8	29	3.5	No grade 3-4 tox.
Tibaldi (20)	II	56	G (3) → DOC (3)	16	43	8	34	4.8	Neutropenia 5.4 Thrombopenia 3.6 Mucositis 3.6 Diarrhea 3.6 Asthenia 9

(a) statistically significant, (b) difference in thrombocytopenia incidence between CT arms was statistically significant (*P* = .0001). (c) asthenia more frequent in the GC arm than VC arm (50% versus 35%, *P* = .015). (d) toxicity evaluated per cycle, (e) worst toxicity per step, G: gemcitabine, IFO: ifosfamide; CDDP: cisplatin; VNR: vinorelbine; CBDCA: carboplatin; PTX: paclitaxel; DOC: docetaxel; RR: response rate; MS: median survival; MPFS: median progression-free survival; NR: not reported; OS: overall survival; PD: progressive disease; SA: single agentNR: not reported.
